# Performance of a Novel Molecular Method in the Diagnosis of Late-Onset Sepsis in Very Low Birth Weight Infants

**DOI:** 10.1371/journal.pone.0136472

**Published:** 2015-08-25

**Authors:** Jonathan Davis, Sharon Christie, Derek Fairley, Peter Coyle, Richard Tubman, Michael D. Shields

**Affiliations:** 1 St Michael’s Hospital, University Hospitals Bristol, Foundation Trust, Bristol, United Kingdom; 2 Belfast Health & Social Care Trust, Belfast, United Kingdom; 3 The Queen’s University of Belfast, Belfast, United Kingdom; University of Florida, UNITED STATES

## Abstract

**Objective:**

To compare the use of a generic molecular assay to ‘standard’ investigations used to assist the diagnosis of late onset bacterial sepsis in very low birth weight infants (VLBW, <1500g).

**Methods:**

VLBW infants, greater than 48 hours of age, who were clinically suspected to have sepsis were investigated using standard tests (full blood count, C-reactive protein (at presentation) and blood culture), in addition, blood was taken for a universal molecular assay (16S rRNA reverse transcriptase PCR) for comparison. Clinical data were recorded during the suspected infection episode. A validated sepsis score (NEO-KISS) was used to retrospectively determine the presence of sepsis (independent of blood culture). The performance of each of the tests were compared by sensitivity, specificity, positive/negative likihood ratios (+/-LR) and postive/negative predictive values (PPV/NPV).

**Results:**

Sixty-five babies with suspected clinical sepsis were prospectively included. The performance indicators are presented with 95% confidence limits. For the detection of bacteria, blood culture had sensitivity of 0.57 (0.34–0.78), specificity of 0.45 (0.30–0.61); +LR of 1.05 (0.66–1.66) and—LR of 0.94 (0.52–1.7); PPV of 33.3 (18.56–50.97) and NPV of 68.97 (49.17–87.72). Serum CRP had sensitivity of 0.92 (0.64–1) and specificity of 0.36 (0.17–0.59); +LR of 1.45 (1–2.1) and-LR of 0.21 (0.03–1.5); PPV of 44.46 (26.6–66.6) and NPV of 88.9 (51.8–99.7). The universal molecular assay had sensitivity of 0.76 (0.53–0.92), specificity of 0.95 (0.85–0.99); +LR of 16.8 (4.2–66.3) and-LR of 0.25 (0.1–0.5); PPV of 88.9 (65.3–98.6) and NPV of 89.4 (76.9–96.5).

**Conclusions:**

In VLBW infants this universal molecular assay performed better in the diagnosis of late onset sepsis (LOS) than blood culture and CRP. Further development is required to explore and improve the performance of the assay in real-time diagnosis.

## Introduction

Late onset infection in the preterm infant is commonly defined as a bacteraemia with an inflammatory response occurring greater than 72 hours of age. Very low birth weight (VLBW) infants are at greater risk of infection because of their developing immune system and the additonal risk factors associated with neonatal intensive care such as the insertion of central long lines [1–3] Incidence of late onset infection has been reported as up to 50% of infants admitted to neonatal intensive care [4,5]. Symptoms and signs of sepsis in VLBW babies can be non-specific. Diagnosis of late onset infection remains a clinical challenge[4–6].

The current ‘gold standard’ for the diagnosis of neonatal blood stream infection is the recovery of organisms by blood culture. There are few published data on the performance of blood cultures in VLBW infants and blood cultures can yield false positive results due to contaminating bacteria from skin. In a post-mortem study involving extremely low birth weight (ELBW) infants, 56 of 111 infants were diagnosed with sepsis after death but 61% of these infants were not considered to be septic during life [7]. In a five-year review of blood cultures in NICU, 48% of positive blood cultures occurred in infants who did not meet criteria for sepsis [8]. C reactive protein (CRP) is also used widely in the diagnosis of preterm infants but is known to be a poor marker especially in the early phase of the disease [9]. There is little data on the performance of blood culture in VLBW babies [10]. In clinical practice these infants are a technical challenge for blood sampling [11]. Other methods have sought to increase the performance of blood culture (multiple site cultures and repeat sampling) but these have been unsuccessful in improving test performance[11,12].

Bacterial detection techniques have evolved and to encompass modern molecular methods, which potentially represent a more rapid, sensitive and specific means of identifying bacteria. Molecular assays that have been studied in neonates include conventional and real time polymerase chain reaction (PCR) assays targeted at universal and specific gene sites [13]. Only a small number of studies have focused on a universal detection method or have included VLBW infants. The first reported use of molecular techniques in neonatal patients was described by Lafrogia et al. in 1997[14]. In this report the use of ‘universal’ 16S rRNA method was described in 33 newborn infants. Since then there have been a number of studies which have explored the use of molecular methods in ‘newborn’ infections [13] using a variety of assays utilizing PCR technology. Few studies have provided a robust exploration of the utility of PCR in a specific population and disease process. A systematic review and meta-analysis [13] reviewed 21 studies which studied bacterial detection (2 focused on the diagnosis of fungus). This review concluded that all the studied molecular methods had a sensitivity of 0.9 (0.78–0.95), meaning that 1 in 10 infants with infection would test negative. In the two studies which specifically included preterm infants the sensitivity for both was <0.9[15,16].

We present a molecular method for the detection of bacteria in VLBW infants presenting with late onset infection. This method is based on reverse transcriptase and probe-based real time PCR amplification of the 16S ribosomal RNA (rRNA) transcript region of the bacterial genome. This technique has not been reported exclusively in VLBW infants before. The aim of this study was to compare the diagnostic ability of this universal assay with CRP and blood culture for the diagnosis of late onset sepsis in VLBW infants.

## Methods

### Patients

We prospectively studied VLBW infants who had been admitted to the neonatal intensive care unit (NICU) of the Royal Jubilee Maternity Hospital, Belfast. The entry point into the study was the clinical suspicion of sepsis, when the infant was more than 48 hours old and was still less than 1500 g in weight. Suspicion of clinical sepsis was determined by the NICU team (medical and nursing). No specific criteria were given to clinical staff as guidance. The attending medical team would us internal judgement and reasoning to determine whether an individual infant required investigation for sepsis. We excluded infants with known sepsis-like inflammatory conditions such as necrotizing enterocolitis (NEC, greater than Bell’s criteria stage II.). This was to ensure a ‘pure sepsis’ population without overlap with NEC.

Clinical and laboratory information were collected from each infant at the time of sample collection. This included patient demographic information, cardiovascular, respiratory, gastrointestinal and neurobehavioral parameters. Laboratory data included total and differential white blood cell count (initial and peak), platelet count (initial and lowest) and C reactive protein concentrations (initial and peak). Other information included the results of any radiological investigation and whether a central line was present. Written informed parental consent was obtained. Ethics approval was granted by the Office of Research Ethic Committees, Northern Ireland (07/NIR01/71).

### Sampling and extraction

Blood was collected by venepuncture or from an indwelling vascular catheter following skin decontamination with an alcohol swab or 0.05% aqueous chlorhexidine solution. Blood sampling was undertaken at the time each infant was reviewed for possible infection. Sampling took place before the administration of antibiotics.

Paediatric BACTEC bottles were used for blood cultures (Becton Dickson and Company, NJ, USA). A blood volume of 0.5 ml was collected in potassium EDTA-coated Vacutainer tubes (Becton Dickson and Company, NJ, USA) for the molecular assay. Samples were stored at 2°C until further processing. Extraction of nuclear material took place within 48 hours of collection. Total nucleic acids were extracted from clinical specimens using the QIAamp DNA Blood Mini Kit (Qiagen, Valencia, CA, USA).

### RT-PCR for 16S rRNA

The 16S rRNA assay was designed for the ABI 7000 sequence detection system (Applied Biosystems, Foster City, CA, USA). The reaction master mix was produced in batches, under conditions designed to reduce the potential for nucleic acid contamination. The mastermix was divided into 1.25 ml aliquots and frozen for use as required throughout the study. The assay was based on a commercial amplification ‘kit’ with the addition of manufactured primers and probes (Superscript III RT-PCR kit: Invitrogen, Carlsbad CA, USA) with combined Primers and Probe (Integrated DNA Technologies, Leuven, Belgium)).

The primers and probes used were based on those as described by Yang et al (2002) [17] and amplified a 161 bp (base pairs) fragment spanning nucleotides 891 to 1051. This is based on the *S*. *aureus* 16S rRNA gene. The primer and probe sequences were P891F–TGGAGCATGTGGTTTAATTCGA and P1033R TGCGGGACTTAACCCAACA—FAM Universal Probe—FAM-CAGGAGCTGACGACARCCATGCA-TAMRA.

The standard volume of master mix used in each reaction was 18μl, with 2μl of extracted clinical sample. The cycling conditions for the described primers were 50°C for 30 minutes, 95°C for 10 minutes followed by 35 cycles of 95°C for 15 seconds and completed at 60°C for one minute. These conditions amplify RNA and DNA, enabling detection of ribosomal RNA molecules, which are present in multiple copies per bacterial cell. No quantification or identification of specific bacteria was undertaken at this stage of the study. In real time PCR a positive reaction is determined by accumulation of fluorescent signal. Positive samples were quoted as cycle threshold (C_*T*_) values, i.e. the number of cycles required for the fluorescent signal to cross the detection threshold. C_*T*_ values are inversely proportional to the amount of target nucleic acid in the sample. The assay had been validated and tested on a panel of gram positive and negative organisms commonly associated with late onset neonatal infection and had been shown to be capable of detection of the common expected organisms.

Clinical samples were amplified along with negative controls (18μl mastermix plus 2μl of nuclease free water). The lowest C_*T*_ value of the negative controls was considered to be the ‘cut off’ for positive samples. Values above this were considered to be negative. Any negative samples were tested using a beta-2-microglobulin PCR assay to determine the presence of viable genetic material.

### Clinical scoring system

We used a non-culture based clinical indicator of sepsis in preterm infants to compare the performance of the new test. This was a combination scoring system based on a German national surveillance scoring system, The Surveillance System nosokomialer Infecktionen fur Fruhgeborene auf Intenivstationen (NEO-KISS) [18]. This system includes clinical, biochemical and haematological criteria. This definition differentiates clinical sepsis, sepsis caused by coagulase negative staphylococcus species and sepsis detected by other organisms. Based on this scoring system, infants at first clinical suspicion of sepsis were classified as NEO-KISS sepsis, yes or no.

NEO-KISS sepsis was defined in this study, as the following: The attending physician instituted antibiotic treatment for 5 days and two of the following clinical criteria were present;

- Fever (>38°C) and/or temperature instability with hypothermia,
- Tachycardia (>200/min) or bradycardia (<80/min),
- Capillary refill time >2 seconds,
- New or increased apnoea >20 seconds,
- Unexplained metabolic acidosis (base excess < -10),
- Hyperglycaemia (serum glucose > 7.5) or
- There were other signs of sepsis including increasing oxygen demand, need for intubation or lethargy.

In addition, one of the following laboratory criteria was required to be present;

- CRP > 2.0 mg/dl,
- Immature to total neutrophil count >0.2,
- Platelets <100/nl or leucocytes <5/nl (18).

These criteria were applied retrospectively without knowledge of the blood culture or 16S rRNA results. A NEO-KISS septic episode was determined to have concluded once the antibiotics had stopped and provided the infant was not re-cultured within 5 days. Only the first sample in the septic episode was studied.

### Analysis

The results are presented as sensitivity/specificity, positive and negative likelihood ratios (+/-LR) and positive/negative predictive values (PPV/NPV) for discrete data with the addition of receiver operating characteristic (ROC) curves for the continuous variables CRP and 16S rRNA. Comparison of CRP between septic and non-septic patients was undertaken with the Mann Whitney U test. Statistical analysis used SPSS 19.0 (IBM, NY, USA) and MedCalc (MedCalc Software bvba, Belgium). The sensitivity, specificity, likelihood ratios and predictive values were calculated from the ROC analysis. These data are calculated from the optimum sensitivity and specificity (performance) for the test in question. The Medcalc software package automatically calculated optimum sensitivity and specificity using the Youden index [19]. This is the maximum distance of the ROC curve from the line of equality (diagonal line on the graph).

## Results

The study was conducted over an 18-month period beginning November 2007. In total, 86 episodes of clinical sepsis in 60 babies (33 males) were recorded and sampled. Nine babies with definite necrotizing enterocolitis (n = 9) were excluded. After excluding repeat testing, 65 samples were analyzed for 16S rRNA. The median gestational age was 26.7 weeks (Interquartile range (IQR): 26–29 weeks) with a median weight 0.98 kg (IQR: 0.77–1.19 kg) and the median age since birth at the time of entry into the study was 15 days (IQR: 6–28 days).

Thirty-six blood cultures were positive and of these 28 were positive for coagulase negative *Staphylococcus* (CONS, *epidermidis* 8, *hominis* 1, *haemolyticus* 1 and 18 were unspecified). Other microbes detected included 4 *Staphylococcus aureus*, 1 *Streptococcus agalactiae*, 1 *Candida albicans* and 2 unspecified mixed growths. Twelve positive blood cultures were classified as ‘NEO-KISS sepsis positive’, 24 as ‘NEO-KISS sepsis negative’ using the described scoring. The bacteria recovered in these groups are described in Table 1.

**Table 1:** Positive blood culture frequency by modified NEO-KISS sepsis score result.

Sepsis Positive (N = 12)	**Coagulase negative *Staphylococcus***	**8**
	Unspecified	6
	*Staphylococcus epidermidis*	1
	*Staphylococcus haemolyticus*	1
	**Mixed growth**	**2**
	***Staphylococcus aureus***	**1**
	***Streptococcus agalactiae***	**1**
Sepsis negative (N = 24)	**Coagulase negative *Staphylococcus***	**20**
	Unspecified	12
	*Staphylococcus epidermidis*	7
	*Staphylococcus hominis*	**1**
	***Staphylococcus aureus***	**3**
	***Candida albicans***	**1**

Positive and negative blood cultures are presented in Table 2. Test performance results are presented with their respective 95% confidence limits. Blood culture had sensitivity of 0.57 (0.34–0.78), specificity of 0.45 (0.30–0.61); +LR of 1.05 (0.66–1.66) and a-LR of 0.94 (0.52–1.7); PPV of 33.3 (18.56–50.97) and NPV 68.97 (49.17–84.72).

**Table 2:** Blood culture result versus modified NEO-KISS sepsis score.

	NEO-KISS Sepsis score		
Blood culture	Positive	Negative	Total
Positive	12	24	36
Negative	9	20	29
Total	21	44	65

The median CRP at presentation in the infants with NEO-KISS sepsis was 19.7 (IQR: 8.35–84.6) and 11.05 (IQR: 3–43.2) in the non NEO-KISS septic infants (p = 0.16). The optimum sensitivity and specificity was determined at a CRP of 5.5 mg/dl. Serum CRP had a sensitivity of 0.92 (0.64–1), specificity of 0.36 (0.17–0.59); +LR of 1.45 (1.0–2.1) and-LR of 0.21 (0.03–1.5); PPV 46.46 (26.6–66.6) and NPV 88.9 (51.8–99.7). The area under the ROC curve for CRP was 0.645 (standard error (SE) 0.1, 0.47–0.8, p = 0.13).

**Fig 1:**
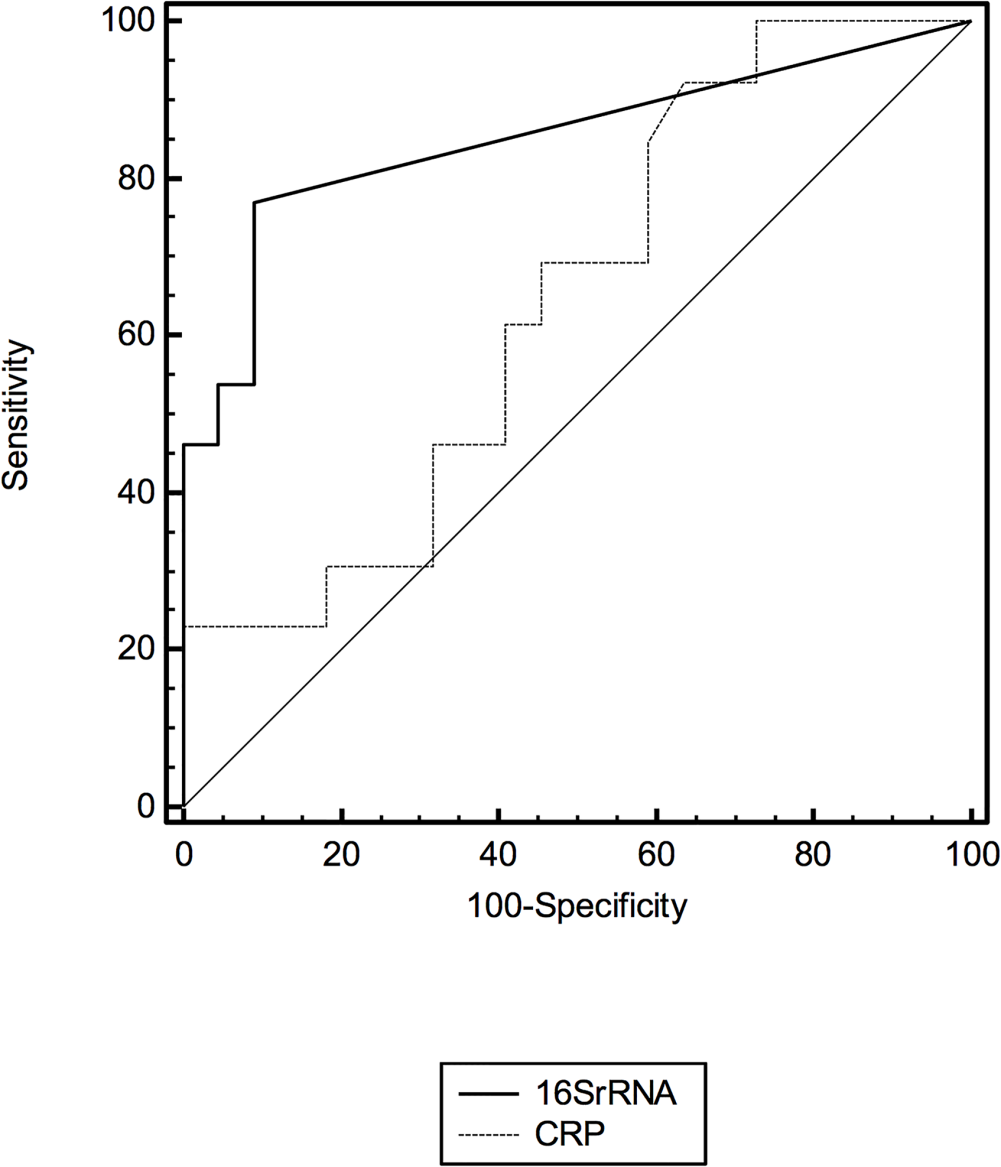
Receiver Operator Characteristic Curve for the 16S rRNA assay and CRP.

The optimum sensitivity and specificity of 16S rRNA was determined if any C_*T*_ value was obtained. The C_*T*_ values for NEO-KISS sepsis positive samples had a median of 21.97 (IQR, 8.49–24.1). The universal RT-PCR 16S rRNA assay had a sensitivity of 0.76 (0.53–0.92), specificity of 0.95 (0.85–0.99), + LR of 16.8 (4.2–66.3) and-LR of 0.25 (0.1–0.5); PPV 88.9 (65.3–98.6) and NPV 89.4 (76.9–96.5). The area under the ROC curve for the molecular assay was 0.86 (SE: 0.05, CI 95%:0.75–0.94, p<0.0001). Receiver operator characteristic curves are presented for the 16S rRNA molecular assay and CRP (Fig 1). The diagnostic performances of the tests are compared below (Table 3).

**Table 3:** Comparison of Blood culture, CRP and 16s rRNA test performance.

	Blood culture	CRP (At presentation)	16S rRNA
Sensitivity	0.57 (0.34–0.78)	0.92 (0.64–1)	0.76(0.53–0.92)
Specificity	0.45 (0.30–0.61)	0.36 (0.17–0.59)	0.95 (0.85–0.99)
+LR	1.05 (0.6–1.66)	1.45 (1–2.1)	16.8(4.2–66.3)
-LR	0.94 (0.52–1.7)	0.21 (0.03–1.5)	0.25 (0.1–0.5)
PPV	33.3 (18.56–50.97)	44.46 (26.6–66.6)	88.9 (65.3–98.6)
NPV	68.97 (49.17–87.72)	88.9 (51.8–99.7)	89.4 (76.9–96.5)

The areas under the receiver operator characteristic curves were compared. The 16S rRNA test area differed to that of the CRP area by 0.21 (SE: 0.11, 95%CI: -0.01–0.43, p = 0.07).

## Discussion

In this study we describe the performance of a real time 16S rRNA assay for the detection of late onset sepsis in a population of VLBW infants. The diagnosis of sepsis in preterm babies is challenging. Blood cultures currently have a central role in the detection of sepsis and are the reference standard for all new detection methods[12]. There are few published studies of blood culture performance in the VLBW population. This casts doubt on the diagnostic efficacy of blood culture in neonatal sepsis. In this study, blood culture had a specificity of 0.57 and sensitivity of 0.45 for the diagnosis of sepsis based on the NEO-KISS score, highlighting its significant limitations as the sole means of diagnosing sepsis in this population.

Blood cultures were positive in 24 babies who had negative NEO-KISS sepsis scores. These infants did not meet the criteria for sepsis. These blood cultures are considered to be false positives. The NEO-KISS score allows for diagnostic determination and comparison of the tests without bias being given to a specific method. We believe this scoring system is the most accurate means of determining the true state of sepsis given the flawed nature of all current methods of determination.

Other tests, such as CRP, have limitations if taken at the time of presentation. CRP was of only moderate value in predicting sepsis (sensitivity 0.92, specificity 0.36). CRP rises more slowly than required for immediate diagnosis but is useful in monitoring the inflammatory response[20].

The positive likelihood ratio (positive LR) is the ratio between the probability of a positive test result given the presence of the disease and the probability of a positive test result given the absence of the disease. In this study the positive LR of 16SrRNA testing was 16.8. This demonstrates that the use of 16S rRNA in this population significantly increases the probability of disease (‘rules in’). The positive likelihood ratios of blood culture and CRP reflect the poor performance of these tests in the VLBW and confirm the superiority of 16S RT-PCR to diagnose sepsis in these infants.

A discussion of the criteria used to determine the presence of sepsis is required. The NEO-KISS is a well-established national surveillance system for nosocomial infection in German NICUs. The surveillance system was developed following an extensive period of validation period. Between 2005 and 2009 the NEO-KISS surveillance group included over 14,321 patients and collected data on neonatal infection, pneumonia and NEC [21]. The group, to maintain the standard of this definition and ensure to the quality of the collected data meets yearly. The definition has been used in a number of studies and is based on the CDC definition of neonatal infection that is a world benchmark for infection reporting but does not itself, have a specific neonatal set of criteria [22] A method of determining the performance of a new test must be compared to a gold standard. In the diagnosis of infection in preterm infants, no such standard truly exists. In this case the most rigorous means of determination is necessary.

Other means of determining neonatal sepsis also exist and as in the NEO-KISS system are used in population surveillance frameworks. These include definitions used in the U.K for the National Neonatal Audit Project [23] and in the U.K and Europe for the NeonIn collaboration [24]. An number of frameworks originate in the U.S. including those used by The National Institute of Child Health and Development (NICHD) [25], the Vermont Oxford Network [26] and the Centres for Disease Control (CDC) [22]. In this study we used the NEO-KISS system as it included criteria that were both clinical and biochemical allowing a culture independent determination of sepsis tailored for VLBW infants[21].

The 16S rRNA molecular assay described in this study demonstrated better performance compared to the other methods of detection with an area under the ROC curve of 0.86 (sensitivity 0.76 and specificity 0.95). The test performance is compared to that described in a recently published systematic review of 23 studies of molecular methods in neonates (sensitivity 0.9 (0.78–0.95) and specificity 0.96 (0.94–0.99)[13]. This review did not include a universal assay targeted in the VLBW population for the detection of LOS. In this meta-analysis sensitivity is separately described for studies in preterm infants (0.79 (16) and 0.69 (15)), LOS studies (0.78 (n = 4)) and in real time PCR studies (0.96 (n = 8) [13]. This review concluded that the specificity and sensitivity of molecular assays are not yet sufficient to be used in place of blood culture. This meta-analysis (13) included a variety of methodologies, patient populations, both early and late onset sepsis, and therefore is not strictly comparable with our study. The only comparable study is that of Makoul et al (2005)[27], who used a *Staphylococcus*-targeted assay in a preterm, VLBW population and found a sensitivity and specificity of 0.57 and 0.95, respectively.

An advantage of a molecular assay is rapidity, as sufficient information is available to potentially stop antibiotics after 3 hours (i.e., sampling, extraction and assay time) from presentation[28]. This would reduce the duration of antibiotics in assay negative infants. This favourably compares to the duration of time required for a blood culture result (2–3 days).

Limitations of this study revolve around that of 16S molecular technology and the relatively poor test sensitivity. The main disadvantage of 16S technology is the potential for reagent contamination with bacterial DNA and the painstaking methods required to prevent this[29]. Contamination was minimized effectively in this study by adherence to good laboratory practice and appropriate preparation of materials. The use of real-time PCR methods also meant that the (almost inevitable) presence of some “background” signal due to contaminating DNA could be corrected for. In this study the presence of bacterial 16S rRNA was used as a general marker of bacterial sepsis, and specific bacterial identification was not attempted. Sequencing of 16S PCR products can be used to obtain more species-specific information within 24 hours [30] As with all current molecular assays, antibiotic sensitivity data require culture-based techniques.

The disappointing test sensitivity is probably related to the known difficulties in the extraction of nuclear material from gram positive bacteria [16]. To further develop and improve assay sensitivity additional protocols for cell wall breakage e.g. freeze-thaw cycles, enzymatic digestion [31] and disposable microfluid chips [32] should be considered.

This molecular assay is more sensitive and specific than blood culture and so provides more definitive information. Smaller test volumes would reduce phlebotomy losses and so aid the preservation of circulating blood volumes in VLBW infants. This assay also has the ability to quantify the degree of bacteraemia and potentially monitor this over time and thus antibiotic response.

## Conclusion

We describe the comparative test performance of two routine and a new RT-PCR 16s based test in the diagnosis of sepsis in VLBW infants. The 16S rRNA assay demonstrated superior performance than blood culture and CRP and may provide a faster, more accurate diagnosis of sepsis in this population of infants.

## Supporting Information

S1 Database (XLS)
